# Fusion and Fission of Genes Define a Metric between Fungal Genomes

**DOI:** 10.1371/journal.pcbi.1000200

**Published:** 2008-10-24

**Authors:** Pascal Durrens, Macha Nikolski, David Sherman

**Affiliations:** MAGNOME Team, INRIA Centre de Recherche Bordeaux - Sud-Ouest, LaBRI (Laboratoire Bordelais de Recherche en Informatique), UMR 5800 CNRS, Domaine Universitaire, Talence Cedex, France; Wellcome Trust Sanger Institute, United Kingdom

## Abstract

Gene fusion and fission events are key mechanisms in the evolution of gene architecture, whose effects are visible in protein architecture when they occur in coding sequences. Until now, the detection of fusion and fission events has been performed at the level of protein sequences with a post facto removal of supernumerary links due to paralogy, and often did not include looking for events defined only in single genomes. We propose a method for the detection of these events, defined on groups of paralogs to compensate for the gene redundancy of eukaryotic genomes, and apply it to the proteomes of 12 fungal species. We collected an inventory of 1,680 elementary fusion and fission events. In half the cases, both composite and element genes are found in the same species. Per-species counts of events correlate with the species genome size, suggesting a random mechanism of occurrence. Some biological functions of the genes involved in fusion and fission events are slightly over- or under-represented. As already noted in previous studies, the genes involved in an event tend to belong to the same functional category. We inferred the position of each event in the evolution tree of the 12 fungal species. The event localization counts for all the segments of the tree provide a metric that depicts the “recombinational” phylogeny among fungi. A possible interpretation of this metric as distance in adaptation space is proposed.

## Author Summary

One consequence of genome remodelling in evolution is the modification of genes, either by fusion with other genes, or by fission into several parts. By tracking the mathematical relations between groups of similar genes, rather than between individual genes, we can paint a global picture of remodelling across many species simultaneously. The strengths of our method are that it allows us to include highly redundant eukaryote genomes, and that it avoids alignment artifacts by representing each group of similar genes by a mathematical model. Applying our method to a set of fungal genomes, we confirmed first that the number of fusion/fission events is correlated with genome size, second that the fusion to fission ratio favors fusions, third that the set of events is not saturated, and fourth that while genes assembled in a fusion tend to have the same biochemical function, there appears to be little bias for the functions that are involved. Indeed, fusion and fission events are landmarks of random remodelling, independent of mutation rate: they define a metric of “recombination distance.” This distance lets us build a genome evolution history of species and may well be a better measure than mutation distance of the process of adaptation.

## Introduction

As the number of complete genome sequences increases, comparative genomics unveils the mechanisms of gene and genome evolution. Duplication, sequence divergence, and recombination are the major mechanisms at work in gene evolution [1]. Recombinational events such as translocation, inversion or segmental duplication can create accidental fusion of DNA sequences associated with different genes, or conversely the fission of a gene into several parts. Potentially, these events can create new genes from already existing parts, or reciprocally shuffle genes into sub-parts across a genome. These rare events participate in the evolutionary history of the species, and must be taken into account in genome rearrangement models.

Methods to inventory gene fusion and fission events on a large biological scale can provide insights about the multimodular architecture of proteins [2],[3],[4], as well as a metric between genomes independently of the mutation rate [2],[5], this work. Computational detection of fusion and fission events uses sequences from several species, usually proteome sequences. This implies that the detection is only performed in the coding regions, a reasonable approximation as non-coding regions evolve faster.

After a recombinational event, gene fusion can occur and is situated either in coding or non-coding sequences. In non-coding sequences, gene fusion can give rise to the misregulation of the expression of a gene now under the control of the *cis*-regulatory sequence of another gene. For instance, the cells in the majority of human prostate cancers bear a gene fusion where the regulatory sequence of the TMPRSS2 gene controls the coding sequence of a transcription factor, either ERG or ETV1, resulting in over-expression of this factor and hence anarchic growth [6]. In coding sequences, gene fusion results in the assembly of a new gene, thereby allowing the emergence of new functions by the accretion of peptide modules into multidomain proteins. As an example, the Tre2 (USP6) oncogene emerged from the fusion of the USP32 and TBC1D3 genes in the hominoid lineage of primates, and it has been proposed that this has contributed to hominoid speciation [7].

Gene fission splits a gene into several parts and can be produced by either recombinational events or single base events, such as frameshift and nonsense mutations. The outcome can be the misregulation of the expression of a gene when a *cis*-regulatory sequence is concerned. Due to the fast evolution of non-coding sequences, the detection of fission events involving such sequences will be out of reach when comparing the genomes of distant species. Loss of continuity in the coding sequences, produced by any of the above events, can give rise to a less complex protein by domain depletion, as, for instance, in the *monkey king* family of genes in *Drosophila* species [8]. Gene fission events can also produce pseudogenes [9].

In completely sequenced prokaryotic genomes, fusions occur more frequently than fissions, and there is no striking bias in the functions of the genes that have undergone these events [5]. The same conclusions hold true in the three kingdoms of the tree of life, by considering the structural domains of the proteins [10]. In mammalian genomes, the close evolutionary distances make it possible to detect fusion and fission events in coding and non-coding sequences; the events between coding sequences involve genes whose protein products have a significant propensity to interact [11]. Fusion events between proteomes have been used to predict protein-protein interactions [12],[13] with some degree of success, in particular metabolic enzymes for which stable protein-protein interactions in one species could be advantageously substituted by the products of fusion events in other species. Altogether, such large-scale comparisons of proteomes revealed that about 4% of the proteins are the products of fused genes and 9% are encoded by genes which are fused in other genomes [2]. These methods work at the level of individual genes, which is an appropriate approach in prokaryotes as the number of duplicated genes is low.

We present here a large scale computation method for detecting gene fusion and fission events in eukaryote genomes, even when they have a noticeable amount of internal gene redundancy. Contrary to the methods published so far, we directly worked at the level of groups of paralogs. We applied the method to the proteomes of a coherent phylogenetic group of species over a large evolutionary range. We chose to focus our study on 12 species covering the phylum of fungi in which a number of complete or near complete genomes are currently available, especially in the group of hemiascomycetes (yeasts). Nonetheless we also chose other ascomycete species as well as basidiomycete and zygomycete species (Table 1). As the evolutionary distances between genomes are large, even inside the group of hemiascomycetes [14], the divergence of non-coding sequences is too high [15] to search for fusion events in them. Since our study is restricted to coding sequences, we employed complete proteomes to track fusion and fission events.

**Table 1:** Proteomes searched.

Phylum	Sub-phylum	Species	Database	Reference
Ascomycota	Hemiascomycota	*Saccharomyces cerevisiae*	SGD	[42]
		*Candida glabrata*	Génolevures	[16]
		*Kluyveromyces lactis*	Génolevures	[16]
		*Eremothecium gossypii*	AGD	[43]
		*Candida albicans*	CandidaDB	[44]
		*Debaryomyces hansenii*	Génolevures	[16]
		*Yarrowia lipolytica*	Génolevures	[16]
	Euascomycota	*Neurospora crassa*	Broad Institute	[45]
		*Aspergillus nidulans*	Broad Institute	[46]
	Archeascomycota	*Schizosaccharomyces pombe*	Wellcome Trust	[47]
			Sanger Institute	
Basidiomycota		*Cryptococcus neoformans*	Stanford Genome	[48]
			Technology Center	
Zygomycota		*Rhizopus oryzae*	Broad Institute	Rhizopus sequencing project (2005)

The species are listed in order from the *Saccharomyces cerevisiae* reference and according to the phylogenetic tree computed by [25]. Proteomes were downloaded as FASTA files.

At first we detected 1103 fusion/fission events, some of them having complex structures which were subsequently decomposed (see Material and Methods), finally giving an inventory of 1680 elementary fusion and fission events in the coding sequences. The number of events in which a species is involved is correlated with the genome size of the species. As some of these genomes are thoroughly annotated, we searched for and could observe slight biases in the biological functions of the genes involved in fusion and fission events compared to those of the other genes. In this phylum, the genes involved in an event tend to belong to the same functional category, a feature already found in prokaryotes [2],[3].

We chose to focus on genome evolution rather than individual domain structure of fusion proteins. Thus we computed the localization of each event in the evolution tree of the 12 fungal species, on the parsimonious assumption that a fusion or fission event happens once during evolutionary history [10]. The weighted counts of events localized in each segment of the phylogenetic tree provided a metric between species, independently of the mutation rate of the genes. From this perspective, it is apparent that some species have undergone massive genome shuffling.

The events relative to the hemiascomycetes will be available in the Genolevures database [16] (http://cbi.labri.fr/Genolevures/) and will be incorporated there in the definitions of protein families.

## Materials and Methods

### Proteomes

The detection of fusion and fission events was performed on the proteomes of species belonging to the group of fungi, with some emphasis in hemiascomycetes as several complete genomes are available. Only complete, or near complete, genomes can provide sets of protein sequence data exhaustive enough to allow precise counts of events. Thus we restricted our study to genomes which were highly covered by sequences (Table 1). When the sequence of a single protein is split into several entries in the proteome file, we deduced that these were sequences of exons and merged these entries to avoid false positive artifacts. A small number of sequences were omitted as they were too short (10 amino-acids or less) to be treated. In some proteomes, a part of the detected events may nonetheless be spurious, due to the quality of sequences and the accuracy of the gene models used to predict introns and coding sequences.

### Detection of Fusion and Fission Events

#### Algorithm

As stated above, the algorithm works at the level of groups of paralogous proteins and extracts simultaneously fusion and fission events in several proteomes.

**Figure 1:**
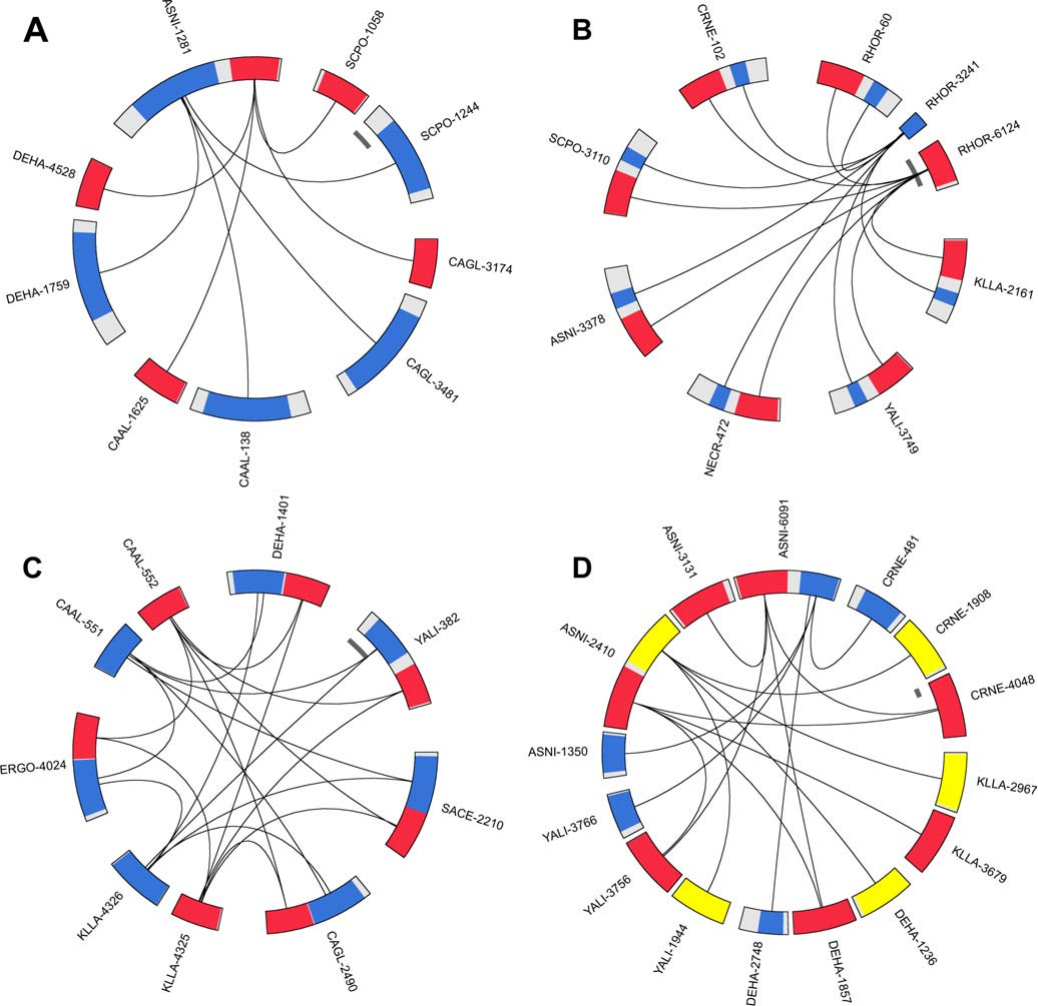
Event examples. (A) Fusion event, (B) Fission event, (C) Multiple event, (D) Complex event, see Algorithm section for definitions. P-groups are drawn to scale and oriented clockwise. Colored areas represent alignment domains, white areas are non-aligned regions. Arcs symbolize relations of similarity between C-groups and E-groups. The inner grey scale bar corresponds to a length of 100 amino acids.

1. As we work on eukaryotic genomes, we expected gene redundancy. Thus, for each proteome, we built a set of paralogous groups (hereafter named *P-groups*), based on sequence similarities (see Software and Parameters below) between proteins. The set of P-groups is thus a partition of the protein set. Note that a P-group may consist only of one protein. Each P-group has a unique name made with an acronym of the species and a number; the acronym is built from the first two letters of the genus and the first two letters of the species, e.g. ASNI-1004 is a P-group of *Aspergillus nidulans*.
2. We then compared all proteomes using an all-against-all comparison of protein sequences. We filtered out the alignment results (see Parameters below) and converted each valid similarity relation between two proteins to a relation between two P-groups. Note that there are relations between P-groups belonging to different proteomes as well as relations between P-groups of the same proteome.
3. The detection of a fusion/fission event requires knowing the extent of the similarity regions between the relevant P-groups. We thus converted each P-group into a multiple alignment, which was in turn converted into a Hidden Markov Model (HMM); in the case of a P-group containing a single sequence, the multiple alignment step was skipped. As HMM-HMM comparisons are very computationally intensive, we restricted these comparisons to the relation between P-groups determined in step (ii), and extracted the coordinates of the aligned regions.
4. We define an *Event* as an *n*-ary relation between P-groups, at least one composite P-group (hereafter named *C-group*) and at least two element P-groups (named *E-groups*), which fulfill three constraints: the E-groups belong to the same proteome, they align on the C-group, the alignment regions have no or reduced overlap on the C-group. Obviously, there could be more than one C-group in an event, as well as more than two E-groups. In [12] the term *component* was used for elements. We considered all the P-groups and their relations, computed in steps (i) and (ii), as a directed graph where P-groups are nodes and each alignment relation is a pair of edges in opposite orientations. Using the above definition of an event, we recursively deleted edges of the graph according to the constraints; when two E-groups had overlapping alignment regions on a C-group, these two E-groups were merged with regard to their relation with the C-group. The events are extracted from the resulting graph as connected components (the term *component* here is used as defined in graph theory).
5. At this stage, a parsimonious interpretation of the events from a phylogenetic point of view, led us to distinguish five types: *Fusion events*, where a single C-group is linked to E-groups issued from at least two species (Figure 1A); *Fission events*, where several C-groups are linked to a set of E-groups coming from a single species (Figure 1B); *Multiple events*, where several C-groups are all linked to several sets of E-groups (Figure 1C); *Undecidable events*, where a single C-group is linked to one set of E-groups coming from a single species, this case can neither be interpreted as a fusion event nor a fission one (not shown); *Complex events*, where several C-groups are linked to different sets of E-groups (Figure 1D).
6. Considering that complex events come from ubiquitous protein domains that are found in several protein architectures, we split these events into events of the four other types, at the expense of doubling some nodes in the graph.

The outcome of this method is an inventory of *elementary* events.

Notes: The algorithm can find events inside a single proteome. Tandem duplication of the same domain within one protein is ignored by the algorithm. The P-groups that are neither C-groups nor E-groups are called *O-groups*, for *Other*.

#### Software and parameters

All proteomes were filtered for their compositionally biased regions with Cast [17].

Sequence comparisons were performed by Blastp [18] with default parameters. We used the same criteria to define a valid similarity between two proteins as those selected for protein family construction [19]:

- a Blastp e-value ≤6.10^−6^,
- a Blastp Positive percentage ≥70%,
- an alignment length ≥70% of the shortest protein.

A P-group only contains proteins with a length greater than or equal to 70% of the length of the largest protein in the P-group.

The accepted overlap for two alignment domains in an C-group is less than or equal to 10% of the total region covered by these domains. We authorized this short overlap as we noticed that a rigorous criterion prevented identification of events already described in *S. cerevisiae*.

P-groups were built starting from multiple alignments done by T-Coffee [20] with default parameters. These multiple alignments were converted into hidden Markov models (HMM) by HHmake from the HHsearch package [21]; the relevant HMMs were then calibrated and compared by HHsearch with default parameters.

Perl scripts using the BioPerl package and the Graph module were written for data handling, result extraction, and ancillary treatments.

Graphical representations of events were computed by Circos [22], which was slightly modified to allow for mock objects (called *spacers*) totally drawn in white.

### Assignment of Biological Functions

We assigned Pfam profiles [23] to P-groups according to the proteins that they contain. We extracted the Pfam identifiers from public databases for a large sample of E-groups and O-groups, in fact all the proteins from *S. cerevisiae*, *C. glabrata*, *K. lactis*, *E. gossypii*, *D. hansenii*, *Y. lipolytica* and *S. pombe*, and we aligned the C-groups to the library of Pfam. Then we converted the Pfam identifiers into GO identifiers [24] through the *pfam2go* conversion file. Finally, we transformed the GO identifiers into high level identifiers in the GO ontology by using the *go2slim* script together with the *yeast GO slim ontology*, thus grouping the identifiers into main categories.

### Fusion/Fission Metric

**Figure 2:**
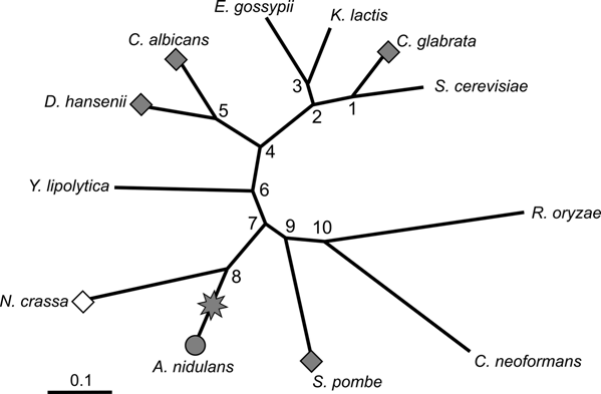
Event localisation. This event is also represented in Figure 1A. *Grey circle*: species having a C-group. *Grey diamond*: species having E-groups as listed in the event. *Open diamond*: species having a P-group similar to one of the E-groups. *Grey star*: parsimonious localization of the event in the phylogeny.

For each event, we mapped the species which contained E-groups or C-groups (Figure 2) onto the phylogenetic tree underlying the 12 species [25]. Under the parsimonious assumption that any event occurred once during evolution, the event should be localised on the tree in one of the edges between the species containing E-groups and the species containing C-groups. Thus, we extrapolated the status of the internal, *i.e.* ancestral, nodes of the tree as either E-group containing node or C-group containing node: (**i**) all internal nodes belonging to a shortest path between two E-group containing species, are extrapolated as E-group containing nodes, *i.e.* the nodes 1, 2, 4, 5, 6, 7, 9 in Figure 2 example; (**ii**) likewise, the status C-group containing node applies to internal nodes between C-group containing nodes, *i.e.* none in the example; (**iii**) the event is inferred to be localised on the shortest path between E-group containing nodes/species and C-group containing nodes/species, *i.e.* either on the edge [node7-node8] or on the edge [node8-*A. nidulans*] in the example; (**iv**) if a given species without status is connected to this last path and if it contains P-groups equivalent to some of the E-groups which defined the event, then the species is assimilated to an E-group containing species and the path can be shortened, *i.e. N. crassa* in the example shortens the path, leaving the [node8-*A. nidulans*] edge as the remaining path; (**v**) each of the *n* remaining edges receives a score of 1/*n*, *i.e.* the [node8-*A. nidulans*] edge receives a score of 1 in the example.

In 4 of the 15 cases of multiple events, the mapping onto the tree brought about internal nodes in which the probable ancestral content in C-groups or E-groups could not be inferred, leading us to suppose that a particular fusion or fission occurred more than once over time. Here, we ordered the involved species in decreasing order of the number of uncertain internal nodes that were resolved when the species was removed. We then used each of these species in this order as the starting point of a shortest path, see preceding paragraph, and removed species until no uncertain internal nodes remained and all of the species were treated. At the end, we identified the minimal number of events necessary to take into account all the species which defined the multiple event and attributed scores to the relevant segments.

## Results

We identified gene fusion/fission events in a coherent phylogenetic group of fungi, where completely sequenced and annotated genomes are avalaible, especially in the hemiascomycete yeasts. Despite this coherency, yeast and fungi encompass large evolutionary distances [26]. We selected 12 species among the fungi phylum tree as representatives, and used our method of event detection on the corresponding proteomes. This method only identified events which occurred inside protein coding genes, but, given the evolutionary distances between species, trying to detect events in intergenic regions would have certainly have been worthless.

**Figure 3:**
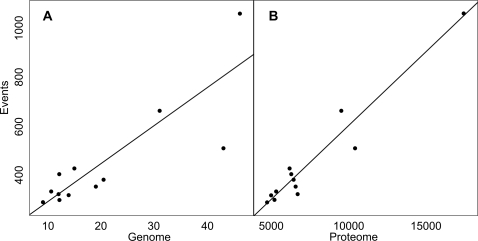
Density of events. Scatterplots of the number of fusion/fission events against (A) genome size in megabases, and (B) proteome size in number of proteins. Straight lines indicate the coefficients determined by bootstrap estimates of robust linear models.

**Figure 4:**
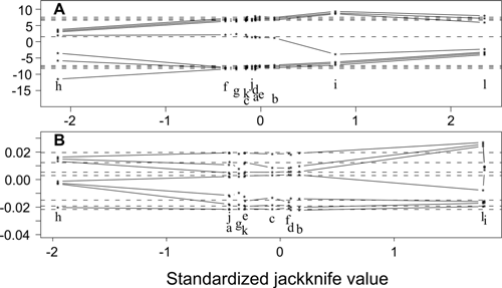
Diagnostic jackknife after bootstrap plots showing sensitivity to individual observations. In particular, NECR (h) tends to decrease the coefficient values. (A) Coefficients for genome size. (B) Coefficients for proteome size (see Figure 3). Observation letters are a *S. cerevisiae*, b *C. glabrata*, c *K. lactis*, d *E. gossypii*, e *C. albicans*, f *D. hansenii*, g *Y. lipolytica*, h *N. crassa*, i *A. nidulans*, j *S. pombe*, k *C. neoformans*, l *R. oryzae*.

We expected gene redundancy since we worked with eukaryotic genomes. If duplicated genes were involved in a fusion / fission event, this event could accordingly be counted several times. To counter this redundancy, we built a set of paralogous groups (*P-groups*) for each proteome. The clustering of several protein sequences inside a P-group was based on sequence similarity and the length of the alignment, to ensure that the proteins shared the same architecture. The set of P-groups is thus a partition of the protein set in a given species (see Dataset S1). Our method is designed to detect events at the level of groups of paralogs (P-groups) and in several proteomes simultaneously (see Material and Methods). The method also finds events which contain E-groups and C-groups belonging to the same species. We detected 1103 events, 176 of them being complex events were subsequently split, giving altogether 1680 elementary events (Table 2 and Dataset S2). These events only involve 12% of the P-groups over all the species, either as E-groups or C-groups. The *Euascomycota* and *Zygomycota* species happen to be the species the most involved in events; these species are those with the larger proteomes and hence the larger genomes. Indeed, we found a correlation between the genome size of a species and the number of events where it appears (Figure 3), a relation also found in a large genome survey [27]. Robust linear models were estimated to predict numbers of events from genome or proteome size, using 5000 bootstrap replications of the Huber regression. Distributions are symmetric overall but not entirely unimodal. Estimated coefficients suggest 15 events per megabase in the genome, or 0.06 events per protein in the proteome. Performing the analysis on a combination of the genome and proteome sizes only slightly improves the model, and is harder to visualize (Figure S1). Jackknife after bootstrap was used to evaluate the sensitivity of the distributions to deletion of individual observations. Species *A. nidulans* and *R. oryzae* would slightly tend to increase the coefficients, while *N. crassa* would tend to decrease it (letters i, l and h, respectively in Figure 4. Generally speaking, *N. crassa* is the most unusual data point and has fewer fusion/fission events than are predicted by the linear model.

**Table 2:** Event statistics.

		Proteome				Event							
Species	Genome Size	Prot.	P	C	E	Inv.	*Fus.*	*Fis.*	*Mul.*	*Und.*	*Exc.*	*Loc.*	*Rec.*
*S. cerevisiae*	12.1	6710	5431	172	312	323	124	151	8	40	54	106	23
*C. glabrata*	12.3	5210	4377	152	251	299	124	138	8	29	36	88	2
*K. lactis*	10.7	5331	4601	150	330	334	127	161	9	37	64	102	15
*E. gossypii*	9.2	4725	4180	146	257	289	124	133	7	25	31	84	3
*C. albicans*	15.0	6165	5152	125	599	428	161	158	11	98	181	144	33
*D. hansenii*	12.2	6277	5114	194	379	405	178	150	11	66	72	123	12
*Y. lipolytica*	20.5	6431	5187	162	401	382	192	149	7	34	45	83	9
*N. crassa*	43.0	10427	9321	213	592	510	245	130	12	123	175	87	21
*A. nidulans*	31.0	9536	7404	514	671	664	298	149	10	207	459	95	171
*S. pombe*	14.0	4990	4078	152	304	318	155	112	7	44	61	68	4
*C. neoformans*	19.1	6578	5502	173	351	354	158	118	10	68	92	84	14
*R. oryzae*	46.1	17461	10349	682	2046	1062	244	184	12	622	800	220	554
**Total**	245.2	89841	70696	2835	5683	1680	376	294	15	995	1665	365	847

Proteome data: *Prot.*: proteins; *P*: P-groups; *C*: C-groups; *E*: E-groups.

Event data: *Inv.*: events where a species is involved; *Fus.*: fusion events; *Fis.*: fission events; *Mul.*: multiple events; *Und.*: undecideable events; *Exc.*: events which no longer exist if a species is removed from the dataset; *Loc.*: events where there are adjacent proteins between E-groups; *Rec.*: events with contain at least C-groups and E-groups of the same species.

Genome sizes are given in Mbases. An event can concern several species, therefore the numbers of events on the Total line are not the sums of the counts per species. All E-groups are counted, even if they can be subsequently merged in events (see Material and Methods).

**Figure 5:**
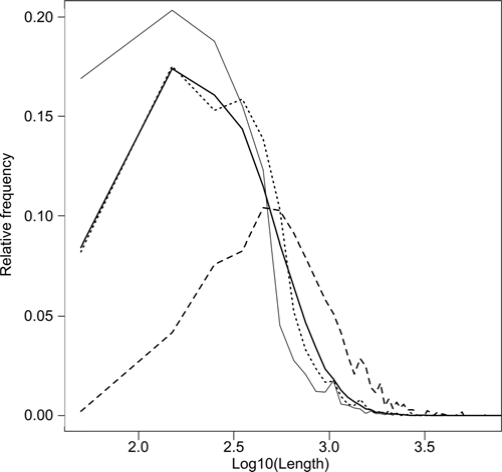
Length of groups and alignments. Dashed line: relative frequencies of C-group lengths (residues). Dotted line: relative frequencies of E-group lengths. Solid bold line: relative frequencies of O-group lengths. Solid thin line: relative frequencies of alignment lengths.

These correlations hold true for the events containing E-groups and C-groups of the same species, about 50% of the events; from a phylogeny angle, these events likely happened recently, that is, after the separation from the last common ancestor with the closest species. The length distributions of the different classes of P-groups and those of the alignments between P-groups used to define events, showed that the C-groups tend to be longer, and that the alignments covered up most of the E-group sequences (Figure 5). The average number of proteins per P-groups is higher in the C-group and E-group subsets compared to the O-groups (2.78, 1.96 and 1.14 respectively), suggesting a higher frequency of duplication for the genes involved in fusion/fission events.

We estimated the value of the fusion over fission ratio to be 1.28 from the number of events classified either as fusion events or as fission events (see Material and Methods), although undecideable events (995 events, Table 2) could not be included in this calculation. This ratio is slightly in favor for fusion events which is in accordance with earlier studies [5],[10].

We then assessed the robustness of the events by removing all the P-groups of one species at a time and then by checking how many events remained (Table 2, column *Exc.*). The number of events exclusive to one species ranged between 31 to 800, suggesting that the set of events is not saturated and that it will increase upon the addition of new species. These numbers, along with the manual curation of the events, indicated that *A. nidulans* and *R. oryzae* genomes were likely to have undergone a large-scale reshuffling.

Our method allowed us to retrieve well-known fusion examples, such as the event involving *S. cerevisiae TRP1*, *TRP3* genes [28] and their homologs in other species (event GFE-1104, see Dataset S2), in which the corresponding polypeptides are separated entities in *Hemiascomycota* and fused in a single protein in *Euascomycota*, *Archeascomycota*, *Basidiomycota* and *Zygomycota*. Another well known example is the one which includes *S. cerevisiae URA2* gene [29]. This very ancient event is thought to have happened before the branching of the fungus phylum, but is still visible as every species kept E-groups and C-groups (event GFE-0970).

Other events can bring information to permit an annotation of ORFs based on the annotation of the fusion product. For instance, in event GFE-0238, the two E-groups contain respectively the uncharacterized ORF *YNR068C* and *YNR069C (BSC5)* ORF of unknown function whereas the *S. cerevisiae* C-group contains *YML111W (BUL2)*, the gene of a “component of the Rsp5p E3-ubiquitin complex, involved in intracellular amino acid permease sorting”, according to Saccharomyces Genome Database annotations.

We tested whether the biological functions of the proteins involved in the events did significantly differ from the functions of the proteins not included in the events. The C-groups were likely to contain several functional domains as they correspond to non overlapping E-groups. We thus chose to predict functional domains using Pfam profiles [23] followed by a conversion into GO terms which were clustered according to the GO-slim “yeast” ontology [24]. We removed the results which mapped to the roots of the ontology, as they were not informative enough; the presented results should therefore be considered as a sample. We followed the same process for E-groups except that, in order to save computation time, we gathered the predicted Pfam identifiers available in public databases for the proteins included in these E-groups. Only eight of the species had this feature (see Material and methods), so again the results should be considered as a sample.

**Figure 6:**
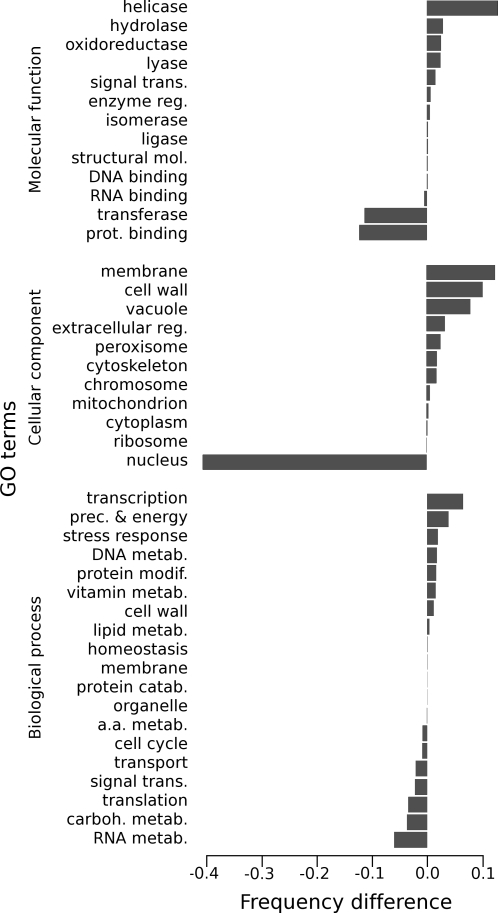
Biological functions tendencies. Differences between GO terms relative frequencies of E-groups and O-groups: positive values mean over-representation of a GO term in E-groups compared to O-groups. Presented GO terms correspond to the main categories of the yeast GO-slim ontology except the roots, (see Text S1 for correspondence between GO term descriptions and their accession numbers).

In the relative frequency differences between E-groups vs. O-groups, only 5 GO slim categories presented a slight over- or under-representation of more than 1% (Figure 6): the nucleus is the under-represented cellular localization of the E-group proteins and the membrane is over represented, proteins classified in “helicase” molecular function are relatively more frequent in E-groups than in O-groups whereas those belonging to “transferase” and “protein binding” molecular functions are less frequent. Some studies reported that most pairs of proteins involved in fusions and with known function, were metabolic enzymes [12],[30]. Another paper [27] indicates receptors and transcription factors to be among the most over-represented functions. As each study was done on a different group of of species, mostly bacteria, and as the set of events is not saturated, it is possible that the discrepancy between these results and ours merely reflects these facts. Moreover, as species may have different ecological constraints and thus different adaptative pressures, it is questionable whether a universal functional bias could be found.

**Figure 7:**
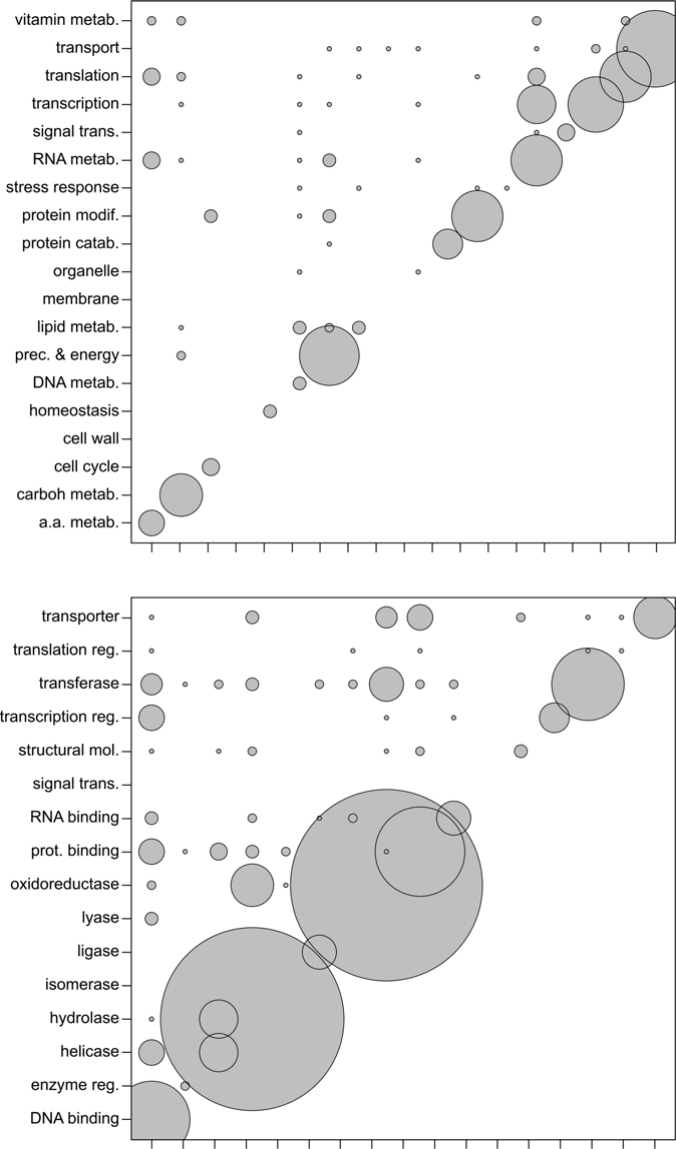
Functional association of fused domains. Pairs of “Yeast GO Slim” terms associated in C-groups (see Methods). Top: Biological process terms. Bottom: Molecular function terms. The GO terms are presented in the same order on both axes: vertically from bottom to top, and horizontally from left to right. The diameter of each circle is proportional to the number of occurrences of the GO term association indicated by the position of its center. (See Text S1 for correspondence between GO term descriptions and their accession numbers.)

The pairs of associated GO-terms, derived from C-groups, were plotted in a square matrix (Figure 7). Pairs were preferentially located on the diagonal of the matrix, indicating that the domains associated in a C-group tend to belong to the same functional category. This point corroborates a similar situation in prokaryotes as found by [2],[3].

Instead of focusing on the individual domain structure of fusion proteins, we chose to consider each event from an evolutionary perspective of genome rearrangement. We thus needed to distinguish two types of event. (**i**) The 365 events where at least one pair of E-groups correspond to adjacent genes on a chromosome, are likely to derive from nonsense or frameshift mutations which transform one coding sequence into two coding sequences or more. We did not take these events into consideration as they *a priori* do not involve genome rearrangement (Table 2, column *Loc.*). (**ii**) The 1315 other events, which contained nonadjacent E-group members, have likely occurred through a recombination event and were therefore the basis of our computation.

**Figure 8:**
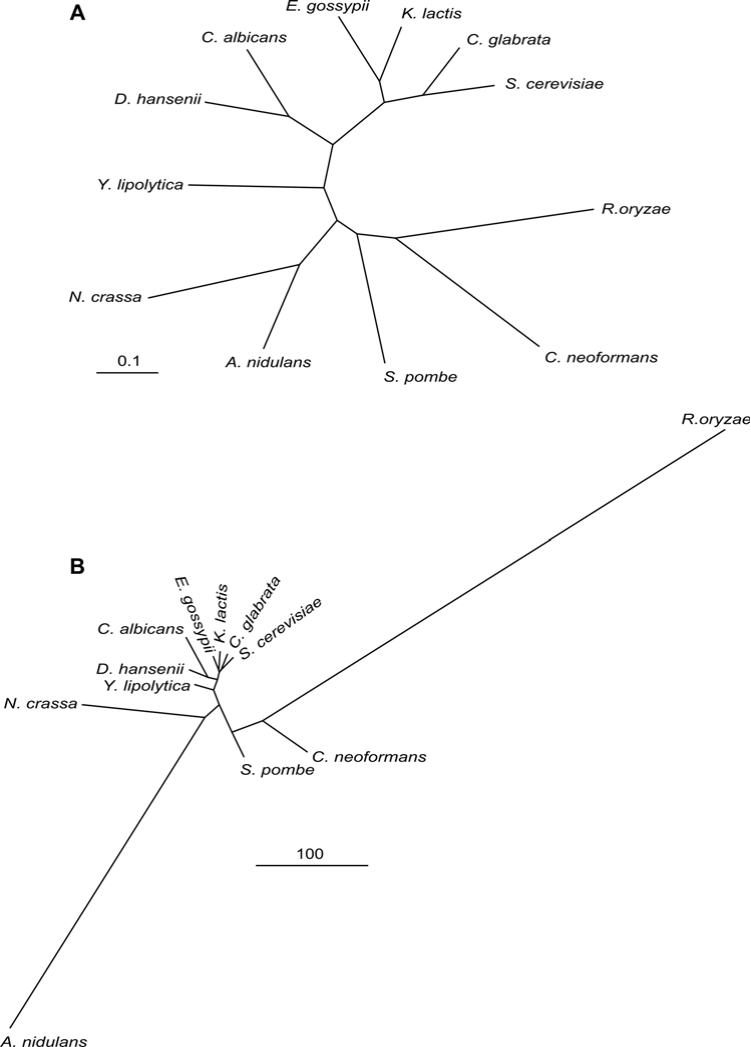
Fungal distance trees. (A) Maximum likelihood tree based on accumulated mutations in 153 universally distributed fungal genes (excerpt from [25]). (B) Rearrangement tree based on the topology of the mutation tree with a modification of branch lengths according to parsimonious localisations of fusion and fission events (see text), the scale bar corresponds to weighted occurences of events.

We then, computed the position of each of these latter events in the evolution tree of the 12 fungal species, derived from the study of [25], with the parsimonious assumption that a fusion or fission event might happen once during evolutionary history [10]. This tree is based on the comparison of the protein sequences translated from families of orthologous genes, and thus was called, in the framework of our study, the “mutation tree” (Figure 8A). Keeping the same topology, we computed the weighted counts of events positioned in each segment of the tree (see Material and Methods), and we changed the length of each tree segment accordingly to make a “recombination tree” (Figure 8B). The use of the event localization weighted counts as a metric dramatically changed the aspect of the tree, making it obvious that some species (*N. crassa*, *A. nidulans* and *R. oryzae*) underwent massive genome shuffling.

## Discussion

Until now, the detection of fusion and fission events has been performed at the level of protein sequences with a post facto removal of supernumerary links due to paralogy. Also, earlier reports often did not look for events only defined in a single genome.

We designed a large scale computation method to detect gene fusion and fission events in eukaryotes genomes taking into account their internal gene redundancy and thus operated at the level of groups of paralogs in the proteomes, named P-groups. The method basically consisted in building a graph of similarity relations between the protein sequences of several species and then pruning this graph according to rules specific to the definition of gene fusion/fission events. The method works simultaneously between every species as well as within species. The output consisted in connected components of the graph, each one defining a fusion/fission event. An event connects “composite” P-groups (C-groups) with “element” P-groups (E-groups). Some of these events could need further splitting into several simpler topologies (elementary events). We distinguished the only four possible topologies, depending on the ratio of E-groups to C-groups in an event.

We applied our method to the kingdom of fungi which covers a large evolutionary range [14], and in which a number of complete or near complete genome sequences are currently available. We chose to focus on a coherent phylogenetic group like fungi, where evolutionary events could be more easily identified, rather than between very distant species, where lifestyle and evolutionary history could make too many events to be immediately instructive. We eventually obtained a set of 1680 elementary fusion and fission events in the coding sequences of 12 fungal species. The number of detected events for a species is related to its genome and proteome size, as it appears to be the case in any species of the tree of life, with few exceptions typically associated with parasitic or infectious lifestyle [5],[27]. The numbers of gene fusion/fission events confirm that these events are relatively rare [2],[5], albeit these numbers are provisional and underestimated as they are not saturated. Thus, the roster of detected events will very likely increase upon the addition of new species into the study.

The fusion/fission ratio of 1.28 was less large than in comparable studies [5],[10], but was still in favor of the fusions. From a phylogeny point of view, we can expect such a tendency, as its beneficial effect would be to permit either the gathering of several biochemical functions into a single polypeptide molecule, thereby reducing the regulation burden of the cell, or the creation of new functions in a scenario which congregates gene duplication, gene fusion and sequence mutation. In the evolution from prokaryotes through lesser eukaryotes and up to higher eukaryotes, a witness of this fusion rate propensity is the observation that proteins have more different domains per protein, along with a larger repertoire of domain combinations [31],[32].

As some of the genomes we used are thoroughly annotated, we could search for biases in the biological functions of the genes involved in fusion and fission events. Only a few were found. Similar findings were reported in other studies [3],[27],[30],[33] although these functions do not appear to be the same in each report. This variation is not surprising since the different works were done on different sets of species, covering one or several kingdoms. In addition, the sets had unequal sizes and, as stated above, the number of events depends on the number of species. Nevertheless, the genes involved in an event in the fungal phylum tend to belong to the same functional category, a feature already found in other contexts [2],[3],[27].

Each event which does not involve adjacent genes on a chromosome, can be interpretated as a landmark of a recombinational event giving rise to gene fusion or fission. We positioned each of such events in the evolution tree of the 12 fungal species on the parsimonious assumption that each happens once during evolutionary history [10]. We only found 7 cases where two independent fissions were necessary for the event to be compatible with the phylogenetic tree (data not shown). For each segment of the tree, the weighted counts of positions provided a metric between the 12 fungal species. This metric is independent of the gene mutation rate, and hence of the “mutation” phylogenetic tree. Rather, the metric depends on another aspect of genome evolution, recombination and gene shuffling. Under this perspective, some species underwent massive genome shuffling, compared to species with more stable chromosome architecture. Other metrics have been proposed to account for a recombitional distance between species, such as a metric based on synteny breakpoints [34]. However this last metric can only be applied on relatively narrow evolutionary distances where synteny exists, such as the vertebrates phylum. In contrast, the fungi phylum encompasses larger distances, for instance even in the Hemiascomycota sub-phylum, synteny blocks shared by *Saccharomyces cerevisiae* and *Yarrowia lipolytica* are too few and far between [14]. The metric we propose deals with traces of recombination events which can persist even if a genome has been totally shuffled.

Several mechanisms of genome recombination could be put forward to explain the appearance of gene fusion and fission. Translocation or inversion can potentially fuse or split genes at their boundaries [35],[36]. Segmental duplication can potentially fuse or split gene at their boundaries, as well as put next to each other exon containing sequences of different origin [37]. Horizontal gene tranfer in bacteria can account for 3% of the fused or split genes [10]. Horizontal gene tranfer is a minor mechanism in fungi [38], but cannot be ruled out as a contributor for fusion/fission events. Partial copies of genes could be inserted in ectopic sites through retrotransposons, potentially creating chimerical genes at the insertion points [39]. Other plausible mechanisms would be transcription mediated gene fusion [40] or retroposition of trans-spliced genes [41]. Whatever the recombination mechanism, it is genetically easier to make a gene fusion than a gene fission [10], because in gene fusion one partner could bring its promoter and the other its terminator, whereas in gene fission, one of the offspring has to come under the control of a new promoter in order to be expressed.

This promoter inheritance and its possible evolutionary divergence will be accessible by testing the genes involved in the events where both C-groups and E-groups exist in the same species, as soon as large scale experimental expression data from the different species will be available. These events, which can be detected by our method, can be considered as evolutionary recent, and thus we may expect a correlation in the patterns of expression of genes from C-groups and those from the E-groups corresponding to the 5′ parts of the C-groups.

During evolutionary time, genomes underwent recombinational events, some of which gave rise to gene fusion or fission, hence new genes and new proteins. Gene fusion and fission can abruptly change the length and composition of a gene, as opposed to point mutations which can alter gene content at a more continuous pace. Evolutionary pressure caused some of the genes produced by fusion or fission to be maintained and propagated until present time. Such genes could thus be considered as participating to the overall fitness and adaptation of a species. If we speculate that a species could be considered as a point in an “adaptation space,” and ecological niches as regions of this space, we could propose the metric we defined as an indirect, or approximate, measure of distance between species in this space. The fact that there is no striking bias in the biological functions of the genes involved in gene fusion or fission, suggests that the recombinational events are basically random. This hypothesis has already been put forward, considering versatility and domain abundance in proteins [32]. Under this consideration, we could also propose that the metric we defined, does not need to be normalized for biological functions, as there is little bias.

The events relative to the hemiascomycetes will be available in the Genolevures database [16] (http://cbi.labri.fr/Genolevures/).

## Supporting Information

Figure S1 Stereo scatterplot and robust linear model of event numbers against both genome and proteome sizes (focus you eyes behind the page until the images merge).
(0.01 MB PNG)

Text S1 GO-terms and their cognate definitions. One line per term: abbreviation as in Figures 6 and 7, GO term number, description.
(0.00 MB TXT)

Dataset S1 P-group compositions. Syntax: Group_name *tab* Protein_name. 1) One line per protein; 2) P-group name is made of an acronym and a number. The acronym is built from the first two letters of the genus followed by the first two letters of the species.
(1.86 MB TDS)

Dataset S2 Event compositions. Syntax: [ Event_name , type  =  type_number *tab* Group_name : : *tab* Merge_name ( List_of_Group_names ) # if necessary : : *tab* Group_name = Group_name # Group-Group relation : : *tab* Merge_name = Group_name # Merge-Group relation ] Type numbers: 1) Undecideable; 2) Fusion; 3) Fission; 4) Multiple. A relation is always written with the E-group on the left side and the C-group on the right.
(0.41 MB TDS)
