# Supplementary figures and images for: Fusion and Fission of Genes Define a Metric between Fungal Genomes
Source: PLoS Comput Biol. 2008 Oct 24;4(10):e1000200. doi: 10.1371/journal.pcbi.1000200 (PMC2557144; doi:10.1371/journal.pcbi.1000200)

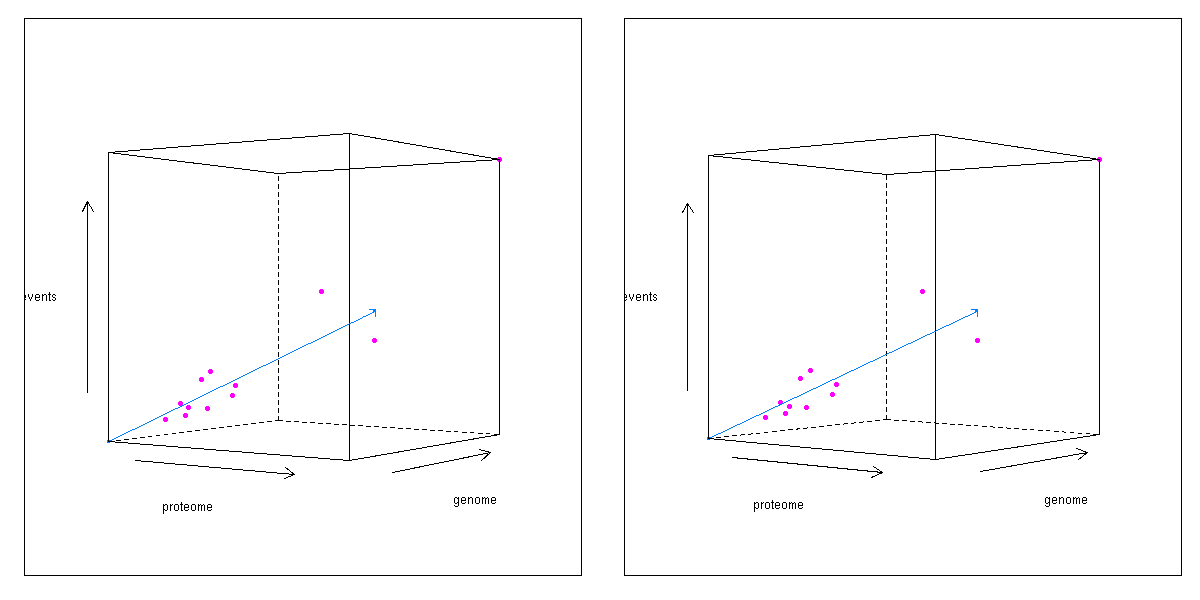

Supplement: Figure S1 — Stereo scatterplot and robust linear model of event numbers against both genome and proteome sizes (focus you eyes behind the page until the images merge). (0.01 MB PNG) [file pcbi.1000200.s001.png]
